# Hierarchical modeling of mechano-chemical dynamics of epithelial sheets across cells and tissue

**DOI:** 10.1038/s41598-021-83396-6

**Published:** 2021-02-18

**Authors:** Yoshifumi Asakura, Yohei Kondo, Kazuhiro Aoki, Honda Naoki

**Affiliations:** 1 Laboratory of Theoretical Biology, Graduate School of Biostudies, Kyoto University, Yoshidakonoecho, Sakyo-ku, Kyoto, 606-8315 Japan; 2 Quantitative Biology Research Group, Exploratory Research Center on Life and Living Systems (ExCELLS), National Institutes of Natural Sciences, 5-1 Higashiyama, Myodaiji-cho, Okazaki, Aichi 444-8787 Japan; 3 Division of Quantitative Biology, National Institute for Basic Biology, National Institutes of Natural Sciences, 5-1 Higashiyama, Myodaiji-cho, Okazaki, Aichi 444-8787 Japan; 4 Department of Basic Biology, School of Life Science, SOKENDAI (The Graduate University for Advanced Studies), 5-1 Higashiyama, Myodaiji-cho, Okazaki, Aichi 444-8787 Japan; 5 Research Center for Dynamic Living Systems, Kyoto University, Sakyo, Kyoto, Kyoto 606-8315 Japan; 6 Theoretical Biology Research Group, Exploratory Research Center on Life and Living Systems (ExCELLS), National Institutes of Natural Sciences, Okazaki, Aichi 444-8787 Japan

**Keywords:** Computational biophysics, Computer modelling, Numerical simulations, Nonlinear dynamics, Cellular motility

## Abstract

Collective cell migration is a fundamental process in embryonic development and tissue homeostasis. This is a macroscopic population-level phenomenon that emerges across hierarchy from microscopic cell-cell interactions; however, the underlying mechanism remains unclear. Here, we addressed this issue by focusing on epithelial collective cell migration, driven by the mechanical force regulated by chemical signals of traveling ERK activation waves, observed in wound healing. We propose a hierarchical mathematical framework for understanding how cells are orchestrated through mechanochemical cell-cell interaction. In this framework, we mathematically transformed a particle-based model at the cellular level into a continuum model at the tissue level. The continuum model described relationships between cell migration and mechanochemical variables, namely, ERK activity gradients, cell density, and velocity field, which could be compared with live-cell imaging data. Through numerical simulations, the continuum model recapitulated the ERK wave-induced collective cell migration in wound healing. We also numerically confirmed a consistency between these two models. Thus, our hierarchical approach offers a new theoretical platform to reveal a causality between macroscopic tissue-level and microscopic cellular-level phenomena. Furthermore, our model is also capable of deriving a theoretical insight on both of mechanical and chemical signals, in the causality of tissue and cellular dynamics.

## Introduction

Collective cell migration in epithelial sheets plays an important role in embryonic development and tissue homeostasis^1^. Such a tissue-level phenomenon must have emerged through interactions between individual cells. However, how the intercellular interactions affect collective cell migration remains unclear, because of the difficulties in understanding causality between the microscopic intercellular process and the macroscopic tissue-level phenomenon. In this study, we aimed to address this issue through computational modeling across the hierarchy between cells and tissues.

Several studies have investigated the molecular basis of chemical and mechanical regulation of cell migration. Recently, it was revealed that actomyosin-generated mechanical force is regulated by chemical cues such as E-cadherin^2^ and Rho GTPases^3,4^ during collective cell migration. In addition, extracellular signal-related kinase (ERK), involved in the MAPK cascade plays an important role in single cell migration in vitro^5^ and in vivo^6,7^, through phosphorylation of myosin light chain (MLC) kinase and focal adhesion kinase.

**Figure 1:**
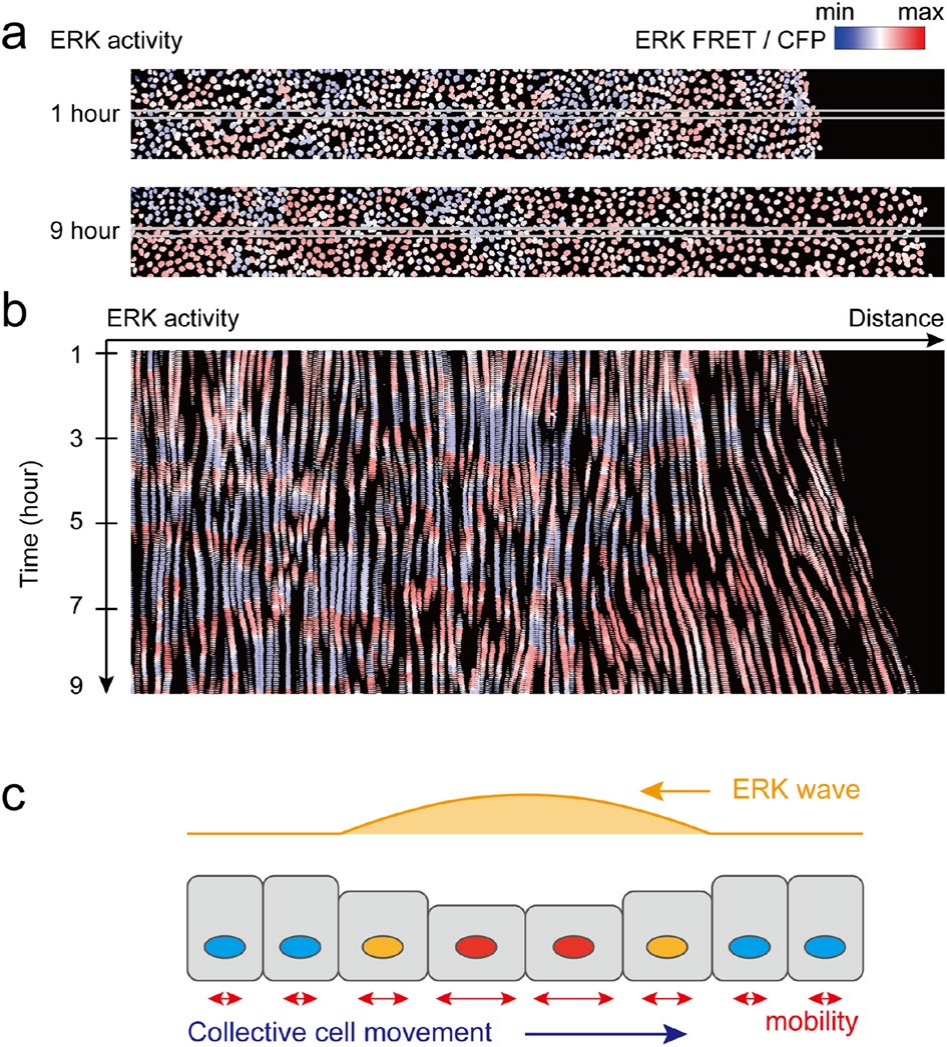
Live imaging of ERK-induced collective cell migration (**a**). Visualization of ERK activity during wound-healing in epithelial MDCK cell sheet expressing the FRET-based biosensor of ERK. Snapshots are presented at 1 h (upper panel) and 9 h (lower panel) after scratching. Red and blue colors indicate high and low ERK activity, respectively. Note that the cytosol is not visualized, since the FRET-based biosensor is localized only in the nucleus^6,10^. (**b**) A kymograph of ERK activity images in a band region of interest indicated by the white box in (**a**). (**c**) A schematic representation of ERK-mediated collective cell migration. Cells migrate toward the opposite direction of the ERK wave. The cellular volume and mobility are increased by ERK activation.

Recently, we reported that collective cell migration during wound healing was mediated by mechano-chemical cellular dynamics involving ERK activation in vivo and in vitro^8,9^. In these previous studies, we experimentally discovered that ERK activation spread in space and time as traveling waves during wound healing, as revealed by live-imaging of ERK activity using a biosensor based on the principle of fluorescence resonance energy transfer (FRET)^6,7,10^. ERK chemical waves spread from the site of injury across the epithelial sheet, and cells collectively move toward the site of injury; this implies that cell migration occurs in a direction opposite to that of ERK waves (Fig. 1, supplemental figure 1, supplemental movie 1). We also showed that cells altered their mechanical properties, including mobility and volume, in response to ERK activation.

**Table 1:** List of parameter values.

Variables	Figs. 2b, 3c–e left, 4a and 5	Figs. 2c, 3c–e middle, 4b	Figs. 2d, 3c–e right, 4c
$\mu _0$	10	10	10
$\alpha$	1.5	1.5	$-$ 0.4
$\beta$	2.5	0	2.5
$k_0$	2	2	2
$R_0$	0.5	0.5	0.5
$\eta$	0.08	0.08	0.08
*r*	1	1	1

To understand how the ERK activation wave is transformed to collective cell migration, we previously developed a particle-based model of an epithelial cell population^8^. This model was formulated as one-dimensionally aligned particles connected by springs, in which particles and springs represent cells and elastic cell–cell interactions, respectively. According to our experiments, we incorporated the effects of ERK signals on cellular mechanical properties, namely ERK-dependent mobility and volume, in the model. Using computer simulations, our model successfully recapitulated the basic property of ERK-mediated collective cell migration, i.e., cells migrate in a direction opposite to that of the ERK traveling wave.

However, the particle-based model could not facilitate a direct comparison of the experimental data with the model, because of the following discrepancies. First, our previous model was implemented in one dimension whereas the epithelial tissue migrated in two dimensions. Second, even in a possible 2D model, it is hard to compare the model with cellular behaviors observed in live-imaging, due to the plastic nature of the tissue. In the tissue, cellular positions are not stably fixed, as is observed in solid particles; it behaves like a fluid in which cellular positions are rearranged in time. Additionally, cells stochastically pass through other cells. Third, cellular responses are heterogeneous; however, our previous model assumed that cells are homogeneous with the same parameter values.

To overcome the aforementioned limitations, we aimed to acquire a coarse-grained model of the two-dimensional epithelial dynamics that averages the heterogeneous properties of individual cells. We propose a hierarchical approach, in which our particle-based model of ERK-mediated collective cell migration was approximately translated into a continuum model. This approach allows us to seamlessly connect hierarchical causality from single-cell behavior to tissue-level dynamics. In the following sections, we describe the particle-based model and its transformation to the continuum model and demonstrate the validity of our hierarchical approach by comparing these two models via numerical simulations.

## Results

### Particle-based model with viscosity

**Figure 2:**
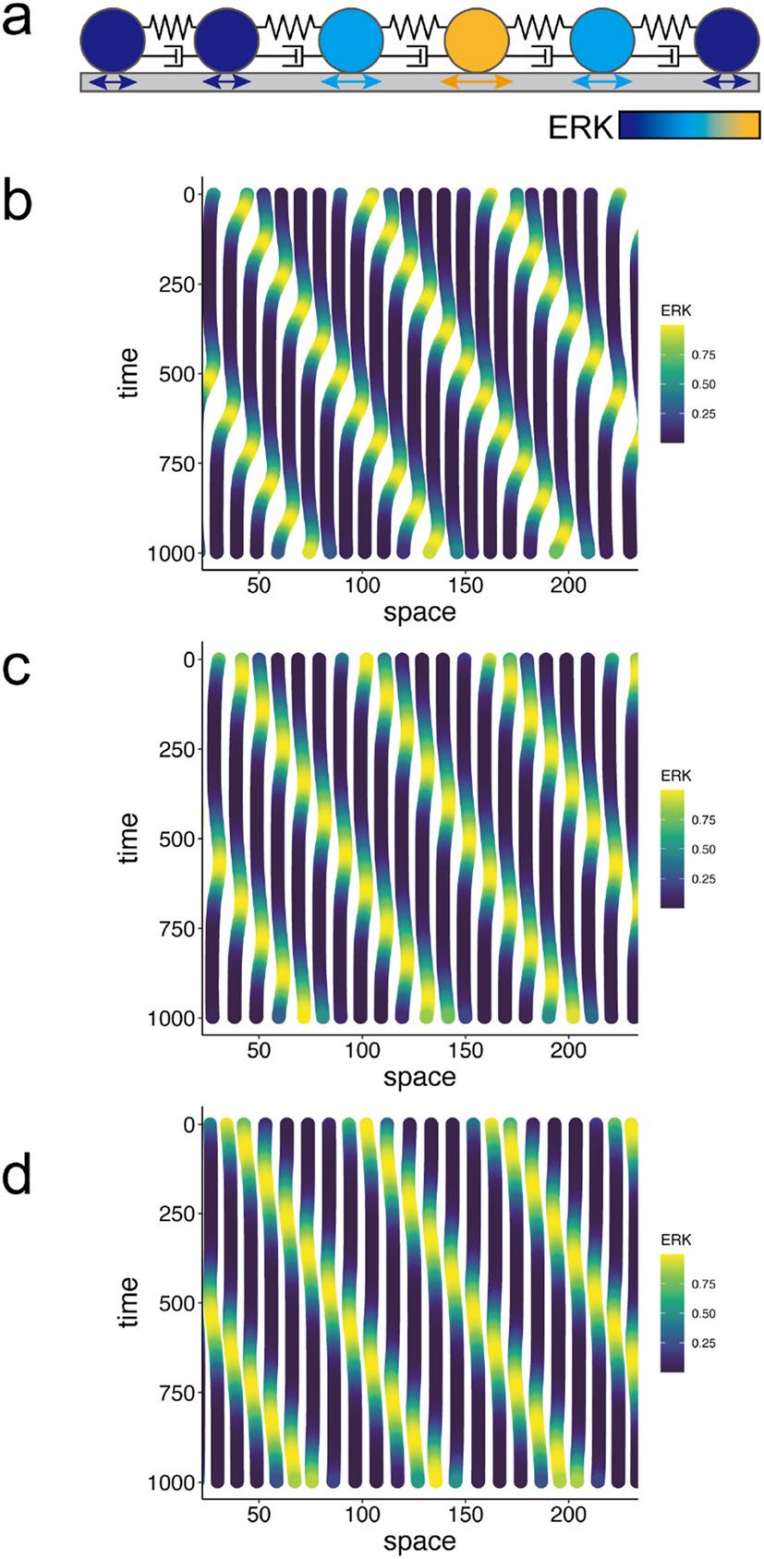
Simulations of the particle-based model in 1D (**a**). Graphical representation of the particle-based model. The centers of cells are illustrated by particles, and these are connected with neighboring cells by springs and dampers representing the elasticity and viscosity of the cytoplasm, respectively. (**b**–**d**). Simulations of ERK-induced collective cell migration with different parameter settings. Each line indicates cellular trajectory with the line color representing ERK activity. Parameters in Table 1 were used. In (**b**), cells migrate toward the opposite direction of the ERK waves. In (**c**), cells show back-and-forth movement during the ERK waves without net displacement. In (**d**), cells migrate toward the same direction of the ERK waves.

Previously, we developed a particle-based model to understand how the ERK traveling wave mediates collective cell migration in epithelial sheets, in which cells adhere to neighboring cells^11–13^ so that they cannot freely migrate due to contact inhibition^14^ but rather mechanically interact each other through repulsive and attractive forces (Fig. 2a). In the model, the epithelial sheet was considered a one-dimensional (1D) series of particles connected through springs, where particles denote the positions of cells and springs represent the elastic property of the cells involved in the membrane, cytoskeleton and adhesion^8^. Thus, the dynamics of the cellular position $x_i$ is described by the following ordinary differential equations (ODEs):

1 $$\begin{aligned} \frac{dx_{i}}{dt}= & {} v_i , \end{aligned}$$

2 $$\begin{aligned} \frac{dv_{i}}{dt}= & {} -\mu _{i}v_{i} - k \{ (R_{i+1} + R_{i}) - (x_{i+1} - x_{i}) \} \nonumber \\&+\, k \{ (R_{i} + R_{i-1}) - (x_{i} - x_{i-1}) \} \nonumber \\&+\, \eta (v_{i+1} - v_{i}) - \eta (v_{i} - v_{i-1}) , \end{aligned}$$

where $x_i$ and $v_i$ indicate the position and velocity of the *i*-th cell, respectively. Parameters $\mu _i, k, R_i$, and $\eta$ indicate the friction coefficient, spring constant, cell radius, and viscosity coefficient, respectively. Because cell size and mobility were upregulated by ERK activation in our previous study^8^, we assumed that cell radius and friction were positively or negatively modulated by ERK activity as

3 $$\begin{aligned} R_i&= R_0 (1 + \alpha ERK_i) , \end{aligned}$$

4 $$\begin{aligned} \mu _i&= \mu _0 \exp (-\beta ERK_i) , \end{aligned}$$

where $R_0$ and $\mu _0$ indicate the basal radius and basal friction of the cells, respectively; $\alpha$ and $\beta$ denote the effect of ERK activity on cellular size and friction, respectively. ERK activity was applied as traveling Gaussian distributions as

5 $$\begin{aligned} ERK_i = A_0 \exp \left[ \frac{-\left\{ x_i - (s_o + ct)\right\} ^2}{2\sigma ^2}\right] , \end{aligned}$$

where $ERK_i, A_0$, $s_o$, *c*, $\sigma ^2$ indicate the ERK activity of the *i*-th cell, amplitude of the ERK wave, initial position of an ERK wave, velocity of the ERK wave, and a positive constant regulating width of the ERK wave, respectively.

Through the simulation, the model showed ERK-induced cell migration, in which the cells migrated in a direction opposite to that of the ERK traveling wave (Fig. 2b); this was consistent with our previous experiments. The cellular migration is driven by imbalance between the friction and the pushing force by the volume expansion, which are caused by the ERK activity wave (supplemental figure 2). Additionally, by changing the simulation parameters, migration in the opposite direction was abolished (Fig. 2c,d). Cancelling the effects of ERK on friction coefficient $\mu _i$ abolished any migration after the passage of an ERK wave (Fig. 2c), and the negative effect of ERK on cell radius $R_i$ caused migration in the same direction as the ERK traveling wave (Fig. 2d). These parameter dependencies of the direction of cellular migration validated our assumption of the effects of ERK on cellular properties expressed in Eqs. (3) and (4).

**Figure 3:**
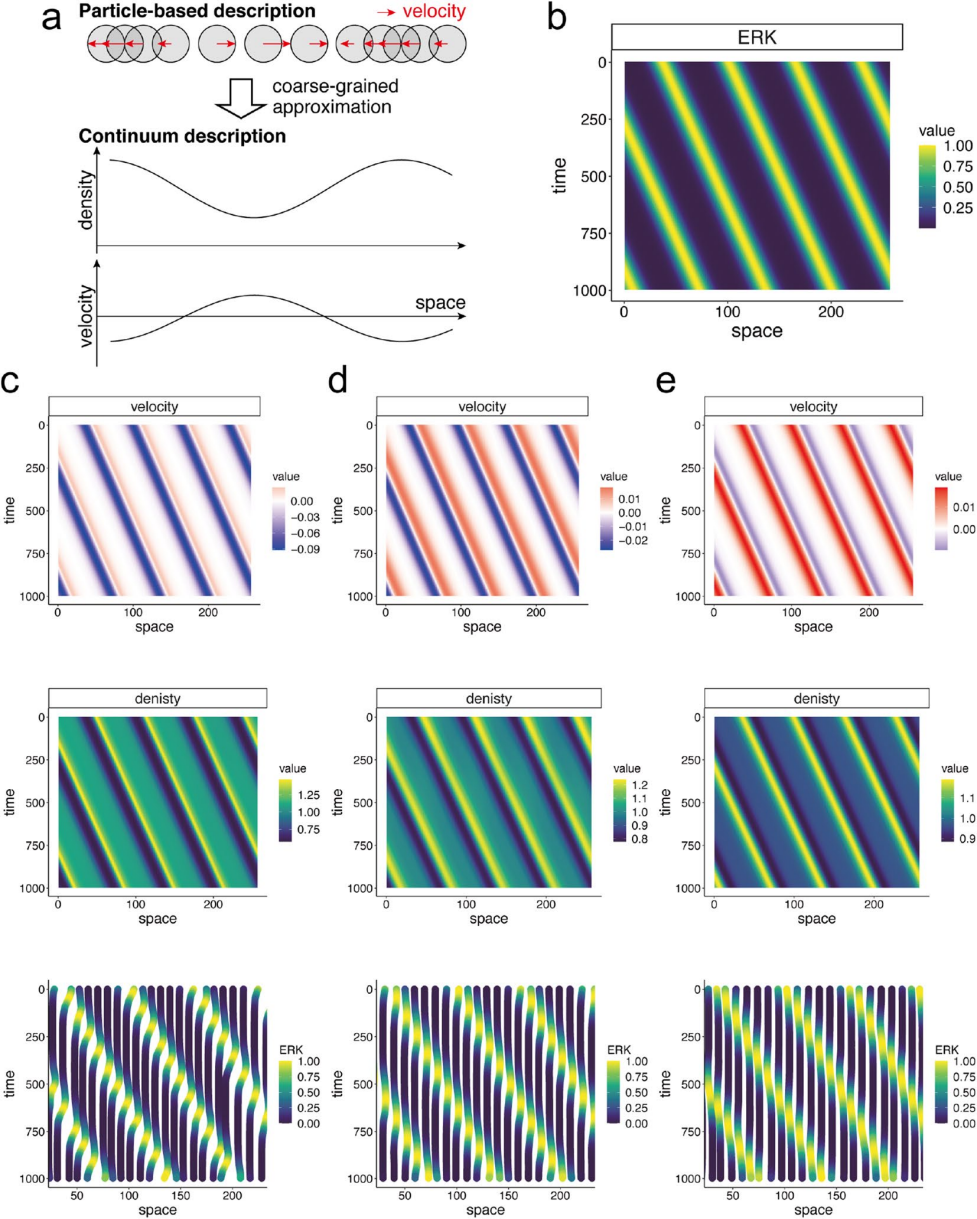
Simulations of the continuum model in 1D (**a**). A schematic representation of the coarse-grained approximation from the particle-based model to continuum model. Migrations of particles, i.e., cells, are represented by density distribution and velocity. (**b**) ERK traveling waves applied in simulations. (**c**–**e**) Simulation results of velocity (upper panel), density (middle panel), and trajectories of the cells (lower panel). Parameters used in (**c**), (**d**) and (**e**) are the same as Fig. 2b–d, respectively.

### Continuum model

We coarse-grained the 1D particle-based model by continuum dynamics approximation (Fig. 3a). We first transformed Eq. (2) into

6 $$\begin{aligned} \frac{dv_i}{dt} = -\mu _{i}v_{i} - (P_{i+1/2} - P_{i-1/2}) + \eta (v_{i+1} - 2v_i + v_{i-1}), \end{aligned}$$

where $P_i = k \left\{ (R_{i+1/2}+R_{i-1/2})-(x_{i+1/2}+x_{i-1/2}) \right\}$. $P_{i-1/2}$ and $P_{i+1/2}$ correspond to the pressure from left and right neighboring cells. Since the cell density is defined as a number of cells within unit distance, the cell density at the middle position between the cells *i* and $i\pm 1$ is calculated by

7 $$\begin{aligned} \rho _{i\pm 1/2} = 1/( x_{i+1}-x_i ) . \end{aligned}$$

In the same way, natural density is defined by $\rho _{i\pm 1/2}^\infty = 1/\left\{ R_{i\pm 1}\left( ERK_{i\pm 1}\right) +R_i\left( ERK_{i}\right) \right\}$. Thus,

8 $$\begin{aligned} P_{i\pm 1/2} = k \left( \frac{1}{\rho ^{\infty }_{i\pm 1/2}} - \frac{1}{\rho _{i\pm 1/2}} \right) . \end{aligned}$$

In the coarse-grained description (6), collective cell migration is interpreted as a continuous flow field, expressed in Lagrangian description in fluid dynamics as follows:

9 $$\begin{aligned} \frac{Dv}{Dt} = -\mu (ERK) v - \frac{1 }{\rho }\frac{\partial P }{\partial x} + \frac{\eta }{\rho ^2}\frac{\partial ^2 v}{\partial x^2} - \frac{\eta }{\rho ^3}\frac{\partial \rho }{\partial x}\frac{\partial v}{\partial x} , \end{aligned}$$

where *v* indicates velocity of the flow field, $P = k \left( 1/\rho ^\infty - 1/\rho \right)$, and $D/Dt= \partial /\partial t + v~{\mathrm {grad}}$ (in this 1D case, $Dv/Dt= \partial v/\partial t + v \partial v/\partial x$, see “Methods” section for details). Because $\rho ^\infty = 1/2R_0 (1 + \alpha ERK)$,

10 $$\begin{aligned} \frac{Dv}{Dt} = -\mu \exp (-\beta ERK)v - 2\alpha kR_0\frac{1}{\rho }\frac{\partial ERK}{\partial x} - \frac{k}{\rho ^3}\frac{\partial \rho }{\partial x} + \frac{\eta }{\rho ^2}\frac{\partial ^2 v}{\partial x^2} - \frac{\eta }{\rho ^3}\frac{\partial \rho }{\partial x}\frac{\partial v}{\partial x}. \end{aligned}$$

Note that *D*/*Dt* is used for the Lagrangian description in which individual cells are tracked and the velocity change is detected as a function of time, whereas $\partial /\partial t$ is used for the Eulerian description in which velocity change is written as a function of space and time.

**Figure 4:**
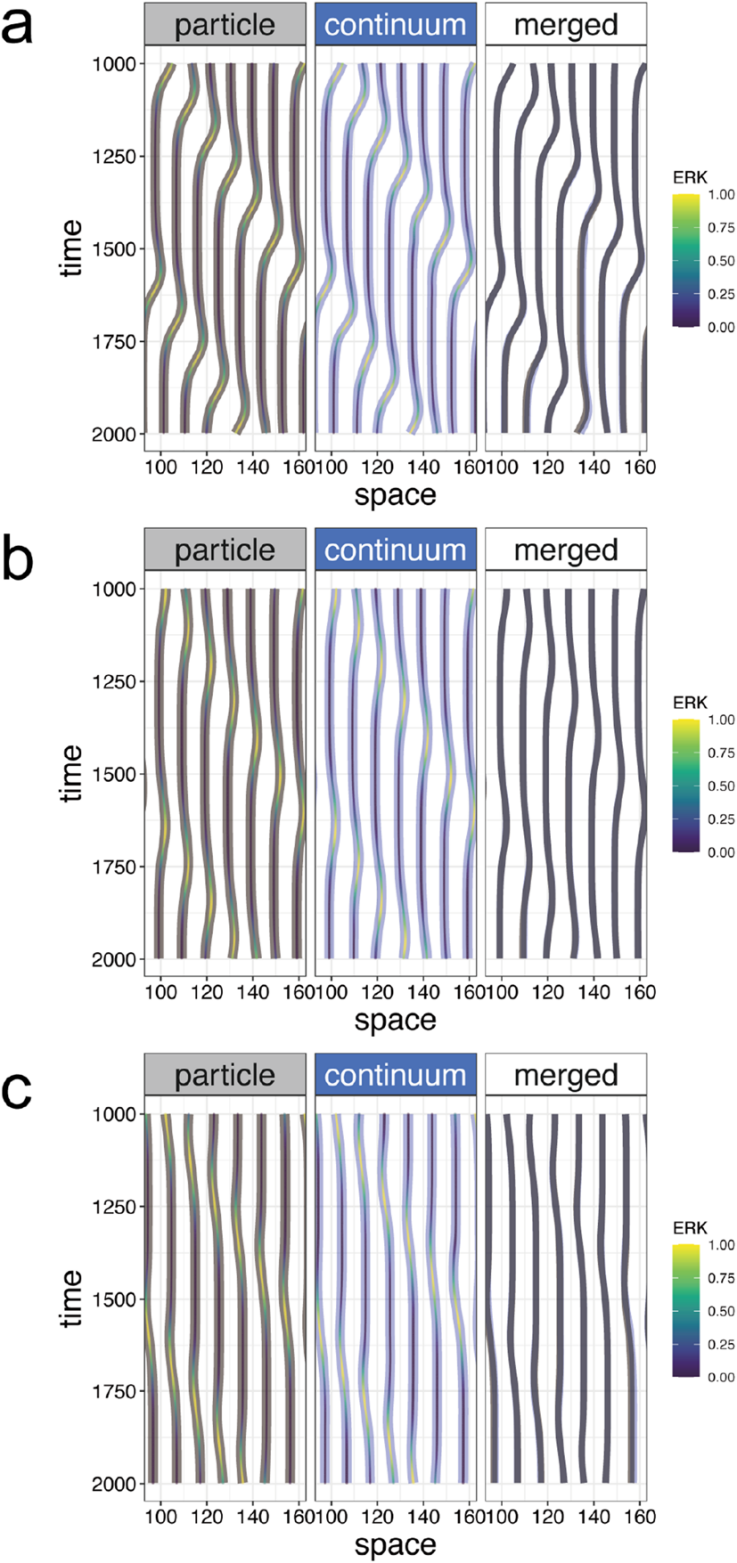
Comparison between the particle-based and continuum models in 1D simulation Comparisons between the migration trajectories simulated by two models with the same parameter values. In the left and center panels, the shaded gray and blue lines indicate trajectories simulated by the particle-based and continuum models, respectively, and the color of the inner lines indicates ERK activity. In the right panels, the shaded gray and blue lines were merged for comparison. Parameters used in (**a**), (**b**), and (**c**) are the same as Fig. 2b–d, respectively.

In contrast to Eq. (6), *v*, *P* and $\rho$ are not indexed by *i*, since we addressed these variables as continuous functions in space and time. The cell density field also has a flux according to Eq. (7) and its dynamics are described by

11 $$\begin{aligned} \frac{D\rho }{Dt} = - \rho \frac{\partial v}{\partial x} . \end{aligned}$$

ERK waves were given the same function as those in the particle-based model (Fig. 3b). With this continuum model, we performed simulations of fields of *v* and $\rho$ in an Euler description with ERK waves that travel in a positive direction on the x-axis. The resulting velocity field showed negative values (Fig. 3c), indicating flow in a direction opposite to that of the ERK waves. The migration in an opposite direction against ERK waves is consistent with our observations in previous experiments. As observed in the particle-based model, velocity fields showed the same direction values in some simulation parameters, as shown in (Fig. 3c–e).

**Figure 5:**
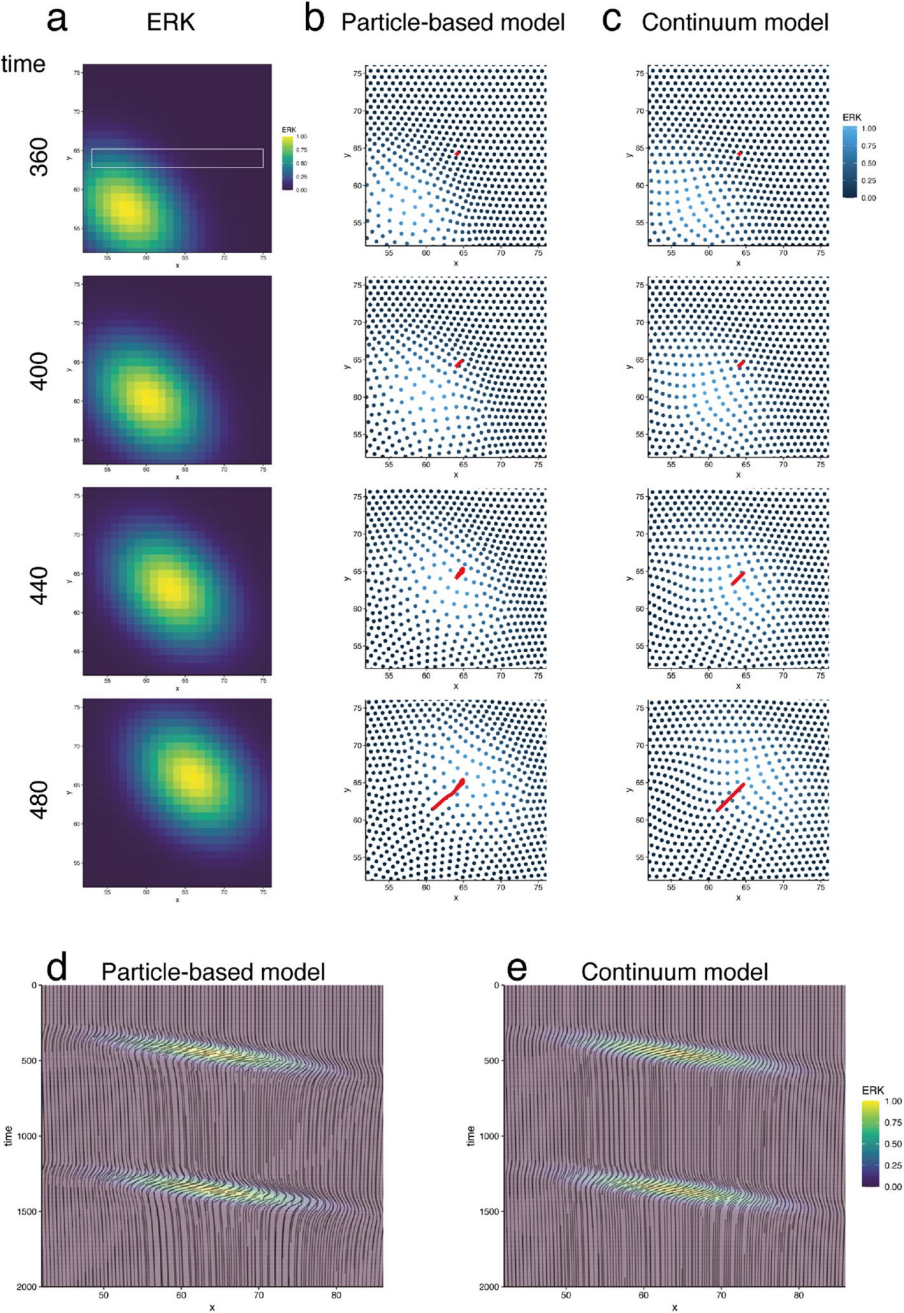
Comparison between the particle-based and continuum models in 2D simulation (**a**). ERK wave applied in the simulations. (**b**,**c**). Simulation results in the particle-based and continuum models. Each dot represents cells in the particle-based model (**b**) and virtual marker simulated in the Lagrange description (**c**). Red lines indicate the trajectories of the cell (**b**) and virtual marker (**c**), which are initially located at the same position. See a supplemental movie for these simulations. (**d**,**e**). Trajectories of the simulated cells in space (*x*)—time coordinates in the particle-based (**d**) and continuum (**e**) models. Cellular migration trajectories within a white box region shown in (**a**).

### Comparison between two models in 1D

To validate our continuum approximation in 1D, we compared the simulation results generated by the two models. To this end, we numerically reconstructed a trajectory of cellular migration, i.e., path line in fluid mechanics, from the simulated velocity field in the continuum model (Fig. 3c–e) (see “Methods” section). It should be noticed that the velocity field in the continuum model represents velocity at time *t* and space *x*, but does not correspond to tracking velocity of specific cells. By comparing the trajectories simulated in our two models, we confirmed that the trajectories generated by the two models were almost the same in various parameters both under symmetric (Fig. 4) and asymmetric (supplemental Fig. 3) ERK waves. We also tested the effect of spatial grid size in the continuum model simulation (supplemental Fig. 4, 5) . As long as the grid size was smaller than ERK wave length, the simulated trajectories were almost the same as ones in particle-based model. These results indicate that our fluid approximation is valid and that the continuum model is able to explain ERK-dependent cell migration.

### Comparison between two models in 2D

We further showed that our continuum model was valid not only in 1D but also in 2D. The 2D particle-based model was extended in 2D. The dynamics of a cell *i* at the position ${\mathbf{x }}_i = (x_i, y_i)$ are described by following ODEs:

12 $$\begin{aligned} \frac{d{\mathbf{x} } _{i}}{dt} = {\mathbf{v }}_{i} , \end{aligned}$$

where ${\mathbf{v }}_i = ({\mathbf{v }}_{x,i},~{\mathbf{v }}_{y,i})$ represents velocity of the cell *i*. Its dynamics are described as

13 $$\begin{aligned} \frac{d{\mathbf{v }}_{i}}{dt} = -\mu _i {\mathbf{v }}_i + \sum _{j} \left( {\mathbf{P }}_{ij} + {\mathbf{V }}_{ij} \right) , \end{aligned}$$

where $\mu _i$ indicates the friction coefficient, which is the same as that in Eq. (4), and ${\mathbf{P }}_{ij}$ and ${\mathbf{V }}_{ij}$ indicates pressure and viscosity, which a neighboring cell *j* applies to the cell *i*. They are expressed as

14 $$\begin{aligned} \mathbf{P }_{ij}&= k\mathbf{e }_{ij} \left\{ |\mathbf{x }_j - \mathbf{x }_i| - (R_i + R_j) \right\} , \end{aligned}$$

15 $$\begin{aligned} \mathbf{V }_{ij}&= \eta (\mathbf{v }_j - \mathbf{v }_i) , \end{aligned}$$

where $k, R_i$, and $\mu _i$ are the spring coefficient, natural length of a cell *i*, and friction coefficient, respectively, and $\mathbf{e }_{ij}$ is a unit vector $(\mathbf{x }_j - \mathbf{x }_i)/|\mathbf{x }_j - \mathbf{x }_i|$ from cell *i* to *j*. The neighboring cells were detected by calculating Voronoi diagrams.

In the 2D continuum model, our model was extended as follows:

16 $$\begin{aligned} \frac{Dv_s}{Dt}&= -\mu \exp (-\beta ERK)v_s - 2\alpha kR_0\frac{1}{\rho }\frac{\partial ERK}{\partial s} - \frac{k}{\rho ^3}\frac{\partial \rho }{\partial s} + \frac{\eta }{\rho ^2}\frac{\partial ^2 v_s}{\partial s^2} - \frac{\eta }{\rho ^3}\frac{\partial \rho }{\partial s}\frac{\partial v_s}{\partial s} , \end{aligned}$$

17 $$\begin{aligned} \frac{D\rho }{Dt}&= - \rho \left( \frac{\partial v_x}{\partial x} + \frac{\partial v_y}{\partial y} \right) , \end{aligned}$$

where $s \in \{x, y\}$. In both models, ERK was described as

18 $$\begin{aligned} ERK_i = A_0 \exp \left[ -\frac{1}{2} \left\{ \mathbf{x }_i - (\mathbf{x }_{o} + \mathbf{c }t)\right\} ^T \Sigma ^{-1} \left\{ \mathbf{x }_i - (\mathbf{x }_{o} + \mathbf{c }t)\right\} \right] , \end{aligned}$$

where $\Sigma$ is a positive symmetric matrix regulating the shape of ERK waves.

With these equations, we conducted numerical simulations. In both models, we confirmed cellular migration in a direction opposite to that of traveling ERK waves (Fig. 5a–c, supplemental movie 2). For comparison between the cellular trajectories simulated by two models in 2D, we plotted cellular trajectories in space-time coordinates, in which cellular trajectories within a 1D band region of interest were depicted (Fig. 5d,e). We noticed that the trajectories in the particle-based model seemed less continuous than those in the continuum model, because the cells were in contact with discrete numbers of neighboring cells, e.g., five or six, and the neighboring pairs were frequently rearranged. Although the trajectories were disconnected because cells leave and enter the band region, the cells showed almost the same migration in a direction opposite to that of the ERK waves in both models. This result showed a clear consistency between the particle-based and continuum models, even in 2D. We further confirmed a consistency between the two models by comparing the simulated trajectories in 2D space (Fig. 5d, e). Taken together, our continuum approximation was validated to recapitulate tissue dynamics, even in 2D.

## Discussion

Here we proposed a hierarchical modeling approach to understand mechano-chemical epithelial dynamics, which acquires causality beyond the hierarchy between the cellular and tissue levels. We showed that the previous particle-based model at the cellular level can be hierarchically transformed into the continuum model to reveal tissue-level epithelial dynamics. Through numerical simulation, we demonstrated the clear consistency of the trajectories simulated by the continuum model and our previous particle model, and our continuum model successfully recapitulated our previous experimental observations. These results indicated the validity of our hierarchical modeling. Thus, our continuum model offers a new theoretical platform to reveal hierarchical regulatory systems between cells and tissues, integrating mechano-chemical signals that determine both cellular and tissue behaviors.

Several coarse-grained models have been developed^15–20^, however, no models for the incorporation of mechanical dynamics with molecular activities that were quantitatively measured by recent imaging techniques with the FRET-based biosensor have been introduced. Due to this lack of integration, mechanistic insights into how intracellular signals, e.g., MAPK and WNT, regulate the mechanical dynamics of tissues and cells have been limited^21^, in spite of the recent progress in imaging techniques. Therefore, our model is the first coarse-grained model to integrate the complex regulatory mechanisms of mechano-chemical tissue dynamics.

Although the epithelial tissue is a 2D sheet, our previous particle-based model was restricted to 1D observations. On the other hand, our continuum model can easily be extended to function as a two-dimensional model and reflects in situ 2D epithelial sheet dynamics observed by imaging experiments. Furthermore, because the continuum model coarse–grains noisy rearrangement of cells, our hierarchical modeling approach presents a new theoretical methodology to understand tissue dynamics without monitoring all cellular behaviors. Therefore, the continuum model is more advantageous than other models at the cellular level, in terms of enabling the interpretation of 2D epithelial sheet dynamics observed by the imaging data^22–24^.

Limitation of our model is that ERK activity was just given as an external input. Hence, how the ERK activity wave is spatially propagated is out of our scope, which is supported by our previous optogenetic experiment. We have shown that artificially-evoked ERK activity wave induced cell migration as observed in wound healing, even under the inhibition of the intercellular ERK activity propagation. Therefore, this experiment suggested that feedback regulation of ERK activity from mechanical force is not essential for collective cell migration. On the other hand, it has been recently reported that the ERK activation wave was linked with mechanical force of cell migration^25^ and mathematically modeled^26^. Another limitation is that the model did not address on a sustained ERK region at the leading edge of the wound (Fig. 1). Actually, during wound healing in vivo^9^ and in vitro with a different experimental setting^25^, the ERK wave was periodically generated and propagated from the wound edge, but the sustained ERK wave was not observed. Therefore, we focused on the cellular migration in response to the ERK waves, but not to the sustained ERK activity.

Since the recent progress in live cell imaging techniques has led to an increase in the number of signaling molecules that can be monitored at the same time, future works need to elucidate the importance of the chemical factors other than ERK. Our continuum model currently takes only one chemical factor into account, and it needs to be further extended to consider two or more factors. Along with the multiple-chemical signal quantifications, recent progress in the quantitative biology field has enabled the measurement of mechanical signals such as pressure and density^27,28^. No methods had been introduced, however, to analyze mechanical signals with canonical molecular biochemical signals at the same time. Our continuum model provides a new theoretical basis to analyze the cellular system, integrating both mechanical and chemical signals.

## Methods

### Detailed derivation of the continuum model

Equation (2) of the particle-based model is rewritten as

19 $$\begin{aligned} \frac{dv_i}{dt}&= -\mu \left( ERK_i\right) v_i - \left( x_{i+1/2}-x_{i-1/2}\right) \frac{P_{i+1/2}-P_{i-1/2}}{x_{i+1/2}-x_{i-1/2}} \nonumber \\&\quad + \eta \left\{ (x_{i+1}-x_{i})\frac{v_{i+1}-v_{i}}{x_{i+1}-x_{i}} - (x_{i}-x_{i-1})\frac{v_{i}-v_{i-1}}{x_{i}-x_{i-1}} \right\} , \end{aligned}$$

where $P_j = k\left\{ (R_{j+1/2} + R_{j-1/2}) - (x_{j+1/2} - x_{j-1/2}) \right\}$ and $P_{i+1/2}$ and $P_{i-1/2}$ correspond to pressure from neighboring cells on the right and left, respectively. Here, we defined the cell density at cell *i* as $\rho _{i} = 1/(x_{i+1/2}-x_{i-1/2})$, where $i+1/2$ represents the middle point index between cell *i* and cell $i+1$. In the same way, the natural cell density at cell *i* is defined by the inverse of the sum of the natural radius of neighboring cells as $\rho ^\infty _{i} = 1/(R_{i+1/2} + R_{i-1/2})$. These definitions lead to $P_{j} = k\left( 1/\rho ^\infty _{j} - 1/\rho _j \right)$. Thus, the Eq. (2) becomes

20 $$\begin{aligned} \frac{dv_i}{dt}&= -\mu \left( ERK_i\right) v_i-\frac{1}{\rho _i} \left. \frac{\partial P}{\partial x}\right| _i \nonumber \\&\quad + \eta \left( x_{i+1/2}-x_{i-1/2}\right) \frac{ \left. \frac{1}{\rho _{i+1/2}}\frac{\partial v}{\partial x}\right| _{i+1/2} - \left. \frac{1}{\rho _{i-1/2}}\frac{\partial v}{\partial x}\right| _{i-1/2}}{x_{i+1/2} - x_{i-1/2}}, \end{aligned}$$

where

$$\begin{aligned} \partial P/\partial x |_{i}&= (P_{i+1/2} - P_{i-1/2})/(x_{i+1/2} - x_{i-1/2}),\\ \partial v /\partial x|_{i}&= (v_{i+1/2} - v_{i-1/2})/(x_{i+1/2} - x_{i-1/2})\\ \end{aligned}$$

According to continuous approximation, i.e. $\lim _{{(x}_{i+1/2}-x_{i-1/2})\rightarrow 0}$, this equation is transformed into

21 $$\begin{aligned} \frac{Dv}{Dt}&= -\mu (ERK)v - \frac{1}{\rho }\frac{\partial P}{\partial x} + \eta \frac{1}{\rho }\frac{\partial }{\partial x} \left( \frac{1}{\rho }\frac{\partial v}{\partial x} \right) \nonumber \\&= -\mu (ERK)v - \frac{1}{\rho }\frac{\partial P}{\partial x} + \eta \left( \frac{1}{\rho ^2}\frac{\partial ^2 v}{\partial x^2} - \frac{1}{\rho ^3}\frac{\partial \rho }{\partial x}\frac{\partial v}{\partial x} \right) , \end{aligned}$$

where *v* indicates continuous function of *x* and *t* as *v*(*x*, *t*), $P(x, t) = k\left\{ 1/\rho ^\infty (x, t) - 1/\rho (x, t) \right\}$, $\rho ^\infty (x, t)=1/2R(ERK(x, t))$ and $Dv/Dt = \partial v/\partial t + v~\partial v/\partial x$. Note that *Dv*/*Dt* indicates Lagrange description of velocity, representing the temporal derivative of the migration velocity of tracking cells. Here, we considered $R(ERK)=R_0 (1+\alpha ERK)$ in the particle-based model, which leads to Eq. (10).

To obtain the continuum dynamics of cell density, we consider the temporal derivative of $\rho _{i}$ referring to the definition $\rho _{i}=1/(x_{i+1/2}-x_{i-1/2})$ as

22 $$\begin{aligned} \frac{d\rho _i}{dt}&= \frac{d}{dt} \left( \frac{1}{x_{i+1/2}-x_{i+1/2}} \right) \nonumber \\&= -\frac{1}{x_{i+1/2} - x_{i-1/2}}\frac{v_{i+1/2} - v_{i-1/2}}{x_{i+1/2} - x_{i-1/2}}. \end{aligned}$$

As described for velocity above, this equation changes to

23 $$\begin{aligned} \frac{d\rho _{i}}{dt} = -\rho _{i} \left. \frac{\partial v}{\partial x} \right| _i. \end{aligned}$$

Again, continuum approximation leads to temporal derivative of density in the Lagrange description:

24 $$\begin{aligned} \frac{D\rho }{Dt} = -\rho \frac{\partial v}{\partial x}. \end{aligned}$$

This equation can be changed in Euler description as

25 $$\begin{aligned} \frac{\partial \rho }{\partial t} = -\frac{\partial }{\partial x}(\rho v). \end{aligned}$$

This equation is well known as the continuity equation, resulting from the law of mass conservation in continuum dynamics. Because it is naturally derived, our continuum approximation was accurate.

Finally, an equation of momentum was derived from Eq. (21). The temporal derivative of momentum is

26 $$\begin{aligned} \frac{\partial p}{\partial t} = v \frac{\partial \rho }{\partial t} + \rho \frac{\partial v}{\partial t} , \end{aligned}$$

where *p* indicates the momentum as $p = \rho v$. Using a general equation between Euler and Lagrange description

27 $$\begin{aligned} \frac{\partial v}{\partial t} + v \frac{\partial v}{\partial x} = \frac{Dv}{Dt} , \end{aligned}$$

and Eqs. (25), (26) changess to

28 $$\begin{aligned} \frac{\partial }{\partial t} p&= -v \frac{\partial (\rho v)}{\partial x} +\rho \left( \frac{Dv}{Dt} - v \frac{\partial v}{\partial x} \right) \end{aligned}$$

29 $$\begin{aligned}&= - \frac{\partial }{\partial x} (v p) + \rho \frac{Dv}{Dt} \end{aligned}$$

30 $$\begin{aligned}&= - \frac{\partial }{\partial x} (v p) - \mu (ERK) p - \frac{\partial P}{\partial x} + \eta \frac{\partial }{\partial x} \left( \frac{1}{\rho }\frac{\partial v}{\partial x} \right) , \end{aligned}$$

where $p = \rho v$ indicates the momentum.

### Alternative derivation of the continuum model

Here, we consider a volume element centered by $x_i$ that contains $\Delta i$ cells and scaled parameters to confirm the model derivation in the previous section by following a procedure established in^20^.

Equation (2) of the particle-based model is rewritten as

31 $$\begin{aligned} m \frac{dv_i}{dt} = -\mu v_i - k \left( h_{i+1/2}-h_{i-1/2} \right) + \eta \left( u_{i + 1/2} - u_{i - 1/2} \right) \end{aligned}$$

where *m* is mass of a cell and $m = 1$ in the Eq. (2), $h_i = (R_{i+1/2} + R_{i-1/2}) - (x_{i+1/2} - x_{i-1/2})$ and $u_{i} = v_{i+1/2} - v_{i-1/2}$. Note that the ERK dependency of $\mu$ is not explicitly written here. This equation is scaled to

32 $$\begin{aligned} {\hat{m}} \frac{dv_i}{dt} = - {\hat{\mu }} v_i - {\hat{k}} \left\{ {\hat{h}} (x_{i + \Delta i/2}) - {\hat{h}}(x_{i - \Delta i/2}) \right\} + {\hat{\eta }} \left\{ {\hat{u}}(x_{i + \Delta i/2}) - {\hat{u}}(x_{i - \Delta i/2}) \right\} , \end{aligned}$$

where ${\hat{m}}, {\hat{\mu }}, {\hat{h}}, {\hat{\eta }}$, and ${\hat{u}}$ are scaled variables for the corresponding variables.

The mass ${\hat{m}}$ is proportional to $\Delta i$ as

33 $$\begin{aligned} {\hat{m}}&= \Delta i ~ m \nonumber \\&= \Delta i . \end{aligned}$$

The friction term is also proportional to $\Delta i$ as

34 $$\begin{aligned} {\hat{\mu }} = \Delta i ~ \mu . \end{aligned}$$

In the pressure term, ${\hat{h}}$ is linearlized by the Taylor expansions with chain rule,

35 $$\begin{aligned} {\hat{h}} \left( x_{i \pm \Delta i/2} \right) = {\hat{h}} ( x_{i} ) \pm {\hat{h}}' \left( x_{i} \right) \left( \frac{1}{2} \frac{\partial x}{\partial i} \Delta i \right) + O(\Delta i ^2) . \end{aligned}$$

Therefore by Eq. (35), the scaled pressure term is

36 $$\begin{aligned} -{\hat{k}} \left\{ {\hat{h}} (x_{i + \Delta i/2}) - {\hat{h}}(x_{i - \Delta i/2}) \right\}&= -{\hat{k}} \frac{\partial x}{\partial i} \frac{\partial {\hat{h}}}{\partial x} \Delta i + O(\Delta i ^2) \nonumber \\&= -k \frac{\partial x}{\partial i} \frac{\partial h}{\partial x} \Delta i + O(\Delta i ^2) , \end{aligned}$$

since ${\hat{k}} = k / \Delta i$, which is the effective spring constant of linearly-connected springs, and ${\hat{h}} = h \Delta i$, which is total displacement of $\Delta i$ cells.

Next, using the Taylor expansions of ${\hat{u}}$ in the same manner as Eqs. (35) and (36), the viscosity term is

37 $$\begin{aligned} {\hat{\eta }} \left\{ {\hat{u}}(x_{i + \Delta i/2}) - {\hat{u}}(x_{i - \Delta i/2}) \right\} = {\hat{\eta }} \frac{\partial x}{\partial i} \frac{\partial }{\partial x} {\hat{u}}(x_i) \Delta i + O(\Delta i ^2) . \end{aligned}$$

Using Taylor expansions of *v* in the same manner as Eqs. (35) and (36) with the Eq. (37),

38 $$\begin{aligned} {\hat{u}}(x_{i})&= {\hat{v}}(x_{i + \Delta i/2}) - {\hat{v}}(x_{i - \Delta i/2}) \nonumber \\&= \frac{\partial x}{\partial i} \frac{\partial v}{\partial x} \Delta i + O(\Delta i ^2) . \end{aligned}$$

Here, ${\hat{v}} = v$ was used since velocity is defined at each location. By Eqs. (37) to (38) with ${\hat{\eta }} = \eta / \Delta i$, which is the effective viscosity constant, the viscosity term becomes

39 $$\begin{aligned} {\hat{\eta }} \left\{ {\hat{u}}(x_{i + \Delta i/2}) - {\hat{u}}(x_{i - \Delta i/2}) \right\} = \eta \frac{\partial x}{\partial i} \frac{\partial }{\partial x} \left( \frac{\partial x}{\partial i} \frac{\partial v}{\partial x} \right) \Delta i + O(\Delta i ^2) . \end{aligned}$$

Then, by ignoring $O(\Delta i ^2)$ terms from Eqs. (33), (34), (36), and (39), the initial Eq. (32) becomes

40 $$\begin{aligned} \frac{dv}{dt} = - \mu \left( ERK\right) v - k \frac{\partial x}{\partial i} \frac{\partial h}{\partial x} + \eta \frac{\partial x}{\partial i} \frac{\partial }{\partial x} \left( \frac{\partial x}{\partial i} \frac{\partial v}{\partial x} \right) , \end{aligned}$$

which is the same as the first line of Eq. (21), since $1 / \rho = \partial x / \partial i$.

Regarding density $\rho$, temporal differentiation of $1 / \rho = \partial x / \partial i$ leads to

41 $$\begin{aligned} \frac{\partial \rho }{\partial t}&= - \rho ^2 \frac{\partial }{\partial t} \frac{\partial x}{\partial i} \nonumber \\&= - \left( \frac{\partial i}{\partial x} \right) ^2 \frac{\partial }{\partial t} \frac{\partial x}{\partial i} \nonumber \\&= - \frac{\partial }{\partial x} \frac{\partial i}{\partial t} \nonumber \\&= - \frac{\partial }{\partial x} \left( \frac{\partial x}{\partial t} \frac{\partial i}{\partial x} \right) , \end{aligned}$$

which is the same as the Eq. (25) since $v = \partial x / \partial t$ and $\rho = \partial i / \partial x$.

### Asymmetric ERK waves

As the asymmetric ERK waves in the simulations, we utilized the skew-normal distribution function which is normalized as its maximum value as 1 was utilized as,

42 $$\begin{aligned} f(x) = \frac{1}{\omega \pi } \exp \left\{ \frac{-(x - \xi )^2}{2 \omega ^2} \int ^{\alpha (\frac{x-\xi }{\omega })}_{-\infty } \exp \left( \frac{-t^2}{2} \right) dt \right\} , \end{aligned}$$

where $\xi , \omega$, and $\alpha$ indicate the location of the distribution, scale, and shape, respectively. This function was implemented in a C++ package “Boost” (https://www.boost.org/).

### Numerical simulation for the particle-based model

Numerical simulations for the particle-based models in 1D and 2D were conducted by the Runge–Kutta method with fourth order accuracy. In the 1D simulation, the initial positions of the particles were equally spaced with unit intervals. In the 2D simulation, the initial positions of the particles were equally spaced on the xy-plane in a hexagonal lattice with unit intervals. Neighboring cells were detected according to the Voronoi diagram with a C++ package qhull^29^.

### Numerical simulation for the continuum model

#### Discretization of the continuum model

For numerical simulations, the continuum PDE model in Euler description was discretized with local Lax–Friedrichs flux^30^. PDEs were generally expressed by

43 $$\begin{aligned} \frac{\partial u}{\partial t} + \frac{\partial f(u)}{\partial x} = S(x,~t), \end{aligned}$$

where $u \in \{ \rho (x,~t), v(x~t) \},~f(u)$ indicates flux, e.g., $f(\rho ) = \rho v$ for cell density and $f(v)=v^{2}/2$ for cell velocity, and *S*(*t*) indicates reaction terms, e.g., $S(t)=0$ for cell density and *S*(*t*) equals to the right-hand-side of Eq. (10). This PDE can be discretized in space and time as

44 $$\begin{aligned} u_j^{\tau + 1} = u_j^{\tau } + \frac{\Delta t}{\Delta x}(f_{j+1/2}^{\tau } - f_{j-1/2}^{\tau }) + S_j^{\tau }\Delta t, \end{aligned}$$

where *j* and $\tau$ denote indices of discretized space and time, respectively; $j\pm 1/2$ corresponds to mid positions between *j* and $j\pm 1$; $\Delta x$ and $\Delta \tau$ indicate the discretized intervals of space and time, respectively; ${\text {s}}_j^\tau$ indicates value at time $\tau \Delta t$ and position $j\Delta x$, $(s\ \in \{u,\ f,\ g\}); u_j^\tau$ represents the spatial average of *u*(*x*) at space *j* as

45 $$\begin{aligned} u_j^{\tau } = \int ^{x_{j+1/2}}_{x_{j-1/2}} u(x,~\tau )dx. \end{aligned}$$

Since $f_{j\pm 1/2}$ indicates flux at the mid positions between *j* and $j\pm 1$, this equation satisfies the conservation of *u*. Here, we considered the discontinuity at $j\pm 1/2$ such that

$$\begin{aligned} \lim _{\delta \rightarrow +0}u \left( (j\pm 1/2)\Delta x + \delta , \tau \Delta t \right) = \left. u^\tau _{j\pm 1/2}\right. ^R, \end{aligned}$$

and

$$\begin{aligned} \lim _{\delta \rightarrow -0}u \left( (j\pm 1/2)\Delta x + \delta , \tau \Delta t \right) = \left. u^\tau _{j\pm 1/2}\right. ^L. \end{aligned}$$

Fluxes at $j\pm 1/2$ were defined by Lax–Friedrichs flux as

46 $$\begin{aligned} f^\tau _{j\pm 1/2} = 1/2\left\{ f(\left. u^\tau _{j\pm 1/2}\right. ^R) + f(\left. u^\tau _{j\pm 1/2}\right. ^L) \right\} - \frac{\gamma }{2}\left( \left. u^\tau _{j\pm 1/2}\right. ^R - \left. u^\tau _{j\pm 1/2}\right. ^L\right) , \end{aligned}$$

where $\gamma = \max _{x}|f(u(x))|$. The first term represents the average flux, whereas the second term indicates artificial diffusion for avoiding numerical stability. $\left. u^\tau _{j\pm 1/2}\right. ^L$ and $\left. u^\tau _{j\pm 1/2}\right. ^R$ in Eq. (46) were calculated by weighted essentially non-oscillatory (WENO) scheme^30,31^ described below.

#### The WENO scheme

We used the fifth-order WENO scheme to compute $\left. u^\tau _{j\pm 1/2}\right. ^L$ and $\left. u^\tau _{j\pm 1/2}\right. ^R$ from values of *u* at the grid points in the stencils $S^L = \{ x_{j-2}, x_{j-1}, x_{j}, x_{j+1}, x_{j+2} \}$ and $S^R = \{ x_{j-1}, x_{j}, x_{j+1}, x_{j+2}, x_{j+3} \}$, respectively. The WENO scheme is a local interpolation method that takes into account the discontinuity of *u*(*x*). Because $u_j$ represents the spatial average of *u*(*x*) over the interval $[x_{j-1/2}, x_{j+1/2}]$, we just know its integral as

47 $$\begin{aligned} U_{j+1/2} = \int ^{x_{j+1/2}}_{x_{0}}u(x)dx = \Delta x \sum ^{j}_{l=0}u_l. \end{aligned}$$

In the WENO reconstruction procedure^31^, values of U were interpolated by the three different stencils of

$$\begin{aligned} S^{(1)}&= \{ x_{j-5/2}, x_{j-3/2}, x_{j-1/2}, x_{j+1/2} \} \\ S^{(2)}&= \{ x_{j-3/2}, x_{j-1/2}, x_{j+1/2}, x_{j+3/2} \} \\ S^{(3)}&= \{ x_{j-1/2}, x_{j+1/2}, x_{j+3/2}, x_{j+5/2} \}, \end{aligned}$$

as third-order polynomials, $P^{(1)}(x), P^{(2)}(x)$ and $P^{(3)}(x)$, respectively. Then, values of *u* were reconstructed by second-order polynomials as $p^{(n)}(x) = dP^{(n)}(x)/dx$, and $u_{j+1/2}$ is approximated to values of $p^{(n)}(x_{j+1/2})$ as

48 $$\begin{aligned} u^{(1)}_{j+1/2}&= \frac{1}{3}u_{j-2} - \frac{7}{6}u_{j-1} +\frac{11}{6}u_{j} \end{aligned}$$

49 $$\begin{aligned} u^{(2)}_{j+1/2}&= -\frac{1}{6}u_{j-1} + \frac{5}{6}u_{j} +\frac{1}{3}u_{j+1} \end{aligned}$$

50 $$\begin{aligned} u^{(3)}_{j+1/2}&= \frac{1}{3}u_{j} + \frac{5}{6}u_{j+1} -\frac{1}{6}u_{j+2}. \end{aligned}$$

Then, $\left. u_{j+1/2}\right. ^L$ was approximated by a convex combination of the three reconstructed $u_{j+1/2}$ as

51 $$\begin{aligned} \left. u_{j+1/2}\right. ^L = w_1 u^{(1)}_{j+1/2} + w_2 u^{(2)}_{j+1/2} + w_3 u^{(3)}_{j+1/2}, \end{aligned}$$

where $\sum _n w_n = 1$ and these weights are modulated in a discontinuity-dependent manner as

52 $$\begin{aligned} w_n = \frac{{\hat{w}}_n}{\sum _k{{\hat{w}}_k}} \end{aligned}$$

with

53 $$\begin{aligned} {\hat{w}}_n = \frac{\lambda _n}{(\epsilon + s_n)^2}, \end{aligned}$$

$\lambda _1=1/10, \lambda _2=6/10, \lambda _3=3/10$ and $\epsilon =10^{-6}$. $s_n$ is smoothness indicator of $p^{\left( n\right) }\left( x\right)$ defined by

54 $$\begin{aligned} s_n = \sum ^m_l \Delta x^{2l-1}\int ^{x_{j+1/2}}_{x_{j-1/2}}\left( \frac{d^l}{dx^l} p^{(n)}(x) \right) ^2 dx, \end{aligned}$$

where *m* is the polynomial degree of *p*(*n*)(*x*) (in this case, $m=2$). The smoothness indicators were formulated by

55 $$\begin{aligned} s_1&= \frac{13}{12}(u_j - 2u_{j+1} + u_{j+2})^2 + \frac{1}{4}(3u_j - 4u_{j+1} + u_{j+2}) \end{aligned}$$

56 $$\begin{aligned} s_2&= \frac{13}{12}(u_{j-1} - 2u_{j} + u_{j+1})^2 + \frac{1}{4}(u_{j-1} - u_{j+1}) \end{aligned}$$

57 $$\begin{aligned} s_2&= \frac{13}{12}(u_{j-2} - 2u_{j-1} + 3u_{j})^2 + \frac{1}{4}(u_{j-2} - 4u_{j-1} + 3u_{j}). \end{aligned}$$

Note that if *u*(*x*) is smooth from $x_{j-1}$ to $x_{j+3}~(i.e., s_1 = s_2 = s_3)$, $\left. u_{j+1/2}\right. ^L$ becomes exactly the same as a polynomial of degree at most four on large stencil *S*. Similarly, $\left. u_{j+1/2}\right. ^R$ can be calculated on stencil $S^R$, but in a mirror symmetric manner with respect to $x_j$ of the above procedure.

#### Numerical simulation in Euler description

The Eq. (43) can be written as a method-of-line ODEs system

58 $$\begin{aligned} \frac{du_j}{dt} = \frac{1}{\Delta x}(f_{j+1/2} - f_{j-1/2}) + S_j \equiv L({\mathbf {u}}_j), \end{aligned}$$

where ${\mathbf {u}}_j = \{ u_{j-2}, u_{j-1}, u_{j}, u_{j+1}, u_{j+2} \}$. These ODEs were temporally integrated by the total variation diminishing (TVD) Runge–Kutta method with third order accuracy^30^ as

59 $$\begin{aligned} u^{(1)}_{j}&= u^{\tau }_{j} + L({\mathbf {u}}^{\tau }_{j})\Delta t \end{aligned}$$

60 $$\begin{aligned} u^{(2)}_{j}&= \frac{3}{4}u^{\tau }_{j} + \frac{1}{4}u^{(1)}_{j} + \frac{1}{4}L({\mathbf {u}}^{(1)}_{j})\Delta t \end{aligned}$$

61 $$\begin{aligned} u^{\tau + 1}_{j}&= \frac{1}{3}u^{\tau }_{j} + \frac{2}{3}u^{(2)}_{j} + \frac{2}{3}L({\mathbf {u}}^{(2)}_{j})\Delta t, \end{aligned}$$

where $${\mathbf {u}}_j^{(k)} = \{ u_{j-2}^{(k)}, u_{j-1}^{(k)}, u_{j}^{(k)}, u_{j+1}^{(k)}, u_{j+2}^{(k)} \}$$.

#### 2D numerical simulation in Euler description

For a 2D simulation in Euler description in the WENO scheme^30^, PDEs were generally expressed by

62 $$\begin{aligned} \frac{\partial u}{\partial t} + \frac{\partial f(u)}{\partial x} + \frac{\partial g(u)}{\partial y} = S(x,~y,~t), \end{aligned}$$

where *S*(*x*,  *y*,  *t*) expresses the reaction term. In the 2D case, we calculated momentum $\rho v_x$ and $\rho v_y$ as *u* in the Eq. (62) instead of calculating $v_x$ and $v_y$ directly. In *x* direction, *u*, *f*(*u*), *g*(*u*) and *S*(*x*,  *y*,  *t*) in the Eq. (62) were

63 $$\begin{aligned} u&= \rho v_x, \end{aligned}$$

64 $$\begin{aligned} f(u)&= v_x u, \end{aligned}$$

65 $$\begin{aligned} g(u)&= v_y u, \end{aligned}$$

66 $$\begin{aligned} S(x,~y,~t)&= \rho \frac{Dv_x}{Dt} . \end{aligned}$$

With Eqs. (63) to (65), each term on the left-hand-side in Eq. (62) becomes

$$\begin{aligned} \frac{\partial u}{\partial t}&= v_x \frac{\partial \rho }{\partial t} + \rho \frac{\partial v_x}{\partial t}, \\ \frac{\partial f(u)}{\partial x}&= v_x \frac{\partial (\rho v_x)}{\partial x} + \rho v_x \frac{\partial v_x}{\partial x}, \\ \frac{\partial g(u)}{\partial y}&= v_x \frac{\partial (\rho v_y)}{\partial y} + \rho v_y \frac{\partial v_x}{\partial y}. \end{aligned}$$

The sum of the first terms on the right-hand-side of the equation was removed as,

$$\begin{aligned} v_x \left( \frac{\partial \rho }{\partial t} + \frac{\partial (\rho v_x)}{\partial x} + \frac{\partial (\rho v_y)}{\partial y} \right) = 0 \end{aligned}$$

since the equation of continuity appears in the brackets. The sum of the second terms becomes

$$\begin{aligned} \rho \left( \frac{\partial v_x}{\partial t} + v_x \frac{\partial v_x}{\partial x} + v_y \frac{\partial v_x}{\partial y} \right) = \rho \frac{D v_x}{Dt}, \end{aligned}$$

which corresponds to the Eq. (66).

Then, the Eq. (62) was discretized in a finite different scheme^30^ as

67 $$\begin{aligned} u_j^{\tau + 1} = u_j^{\tau } + \frac{\Delta t}{\Delta x}(f_{j+1/2}^{\tau } - f_{j-1/2}^{\tau }) + \frac{\Delta t}{\Delta x}(g_{j+1/2}^{\tau } - g_{j-1/2}^{\tau }) + S_j^{\tau }\Delta t. \end{aligned}$$

Note that in the finite different scheme, each of the numerical fluxes $f_{j \pm 1/2}^{\tau }$ and $g_{j \pm 1/2}^{\tau }$ can be computed independently. Therefore, the numerical fluxes were computed in the same way as in the 1D case.

#### Numerical simulation in Lagrange description

After obtaining the field value $v\left( x,t\right)$, Lagrange-described value $x(x_0,T)$ at time T is calculated by

68 $$\begin{aligned} \frac{dx}{dt} = v(x, t) , \end{aligned}$$

by referring $v\left( x,t\right)$. The calculations were conducted using the Runge–Kutta method with fourth order accuracy in 1D simulations, and on an implicit Runge–Kutta method of the Radau IIA family of order five in 2D simulations. Initial positions of particles on the tissue were determined as the same initial positions as those in the particle model simulations.

## Supplementary Information

Supplementary Information.

Supplementary Video 1.

Supplementary Video 2.

## Data Availability

All relevant data have been provided within the paper.

## Code availability

The codes are available in GitHub (https://github.com/asakura-yoshifumi/publication20200818).

## References

[1] Friedl P, Gilmour D (2009). Collective cell migration in morphogenesis, regeneration and cancer. Nat. Rev. Mol. Cell Biol..

[2] Cai D (2014). Mechanical feedback through e-cadherin promotes direction sensing during collective cell migration. Cell.

[3] Hidalgo-Carcedo C (2010). Collective cell migration requires suppression of actomyosin at cell–cell contacts mediated by DDR1 and the cell polarity regulators Par3 and Par6. Nat. Cell Biol..

[4] Reffay M (2014). Interplay of RhoA and mechanical forces in collective cell migration driven by leader cells. Nat. Cell Biol..

[5] Huang C, Jacobson K, Schaller MD (2004). MAP kinases and cell migration. J. Cell Sci..

[6] Kamioka Y (2012). Live imaging of protein kinase activities in transgenic mice expressing FRET. Biosensors.

[7] Mizuno R (2014). In vivo imaging reveals PKA regulation of ERK activity during neutrophil recruitment to inflamed intestines. J. Exp. Med..

[8] Aoki K (2017). Propagating wave of ERK activation orients collective cell migration. Dev. Cell.

[9] Hiratsuka T (2015). Intercellular propagation of extracellular signal-regulated kinase activation revealed by in vivo imaging of mouse skin. Elife.

[10] Komatsu N (2011). Development of an optimized backbone of FRET biosensors for kinases and GTPases. Mol. Biol. Cell.

[11] Papusheva E, Heisenberg C (2010). Spatial organization of adhesion: force-dependent regulation and function in tissue morphogenesis. EMBO J..

[12] Takeichi M (2014). Dynamic contacts: rearranging adherens junctions to drive epithelial remodelling. Nat. Rev. Mol. Cell Biol..

[13] Bosch-Fortea M, Martín-Belmonte F (2018). Mechanosensitive adhesion complexes in epithelial architecture and cancer onset. Curr. Opin. Cell Biol..

[14] Kawabata N, Matsuda M (2016). Cell density-dependent increase in tyrosine-monophosphorylated ERK2 in MDCK cells expressing active Ras or Raf. PLOS ONE.

[15] Brodland GW (2003). Do lamellipodia have the mechanical capacity to drive convergent extension?. Int. J. Dev. Biol..

[16] Fozard J, Byrne H, Jensen O, King J (2009). Continuum approximations of individual-based models for epithelial monolayers. Math. Med. Biol..

[17] Ishihara S, Marcq P, Sugimura K (2017). From cells to tissue: a continuum model of epithelial mechanics. Phys. Rev. E.

[18] Murisic N, Hakim V, Kevrekidis IG, Shvartsman SY, Audoly B (2015). From discrete to continuum models of three-dimensional deformations in epithelial sheets. Biophys. J..

[19] Turner S (2005). Using cell potential energy to model the dynamics of adhesive biological cells. Phys. Rev. E.

[20] Murray PJ, Edwards CM, Tindall MJ, Maini PK (2012). Classifying general nonlinear force laws in cell-based models via the continuum limit. Phys. Rev. E.

[21] Avvisato CL (2007). Mechanical force modulates global gene expression and $$\beta $$-catenin signaling in colon cancer cells. J. Cell Sci..

[22] Honda H (1983). Geometrical models for cells in tissues. Int. Rev. Cytol..

[23] Nonomura M (2012). Study on multicellular systems using a phase field model. PLoS ONE.

[24] Yamao M, Naoki H, Ishii S (2011). Multi-cellular logistics of collective cell migration. PLoS ONE.

[25] Hino N (2020). ERK-mediated mechanochemical waves direct collective cell polarization. Dev. Cell.

[26] Boocock, D., Hino, N., Ruzickova, N., Hirashima, T. & Hannezo, E. Theory of mechanochemical patterning and optimal migration in cell monolayers. *Nat. Phys.* (2020). 10.1038/s41567-020-01037-7.

[27] Tambe DT (2011). Collective cell guidance by cooperative intercellular forces. Nat. Mater..

[28] Trepat X, Wasserman M, Angelini T (2009). Physical forces during collective cell migration. Nat. Phys..

[29] Barber CB, Dobkin DP, Huhdanpaa H (1996). The quickhull algorithm for convex hulls. ACM Trans. Math. Softw. (TOMS).

[30] Shu C-W (2009). High order weighted essentially nonoscillatory schemes for convection dominated problems. SIAM Rev..

[31] Cockburn, B., Shu, C.-W., Johnson, C. & Tadmor, E. Advanced Numerical Approximation of Nonlinear Hyperbolic Equations, Lectures given at the 2nd Session of the Centro Internazionale Matematico Estivo (C.I.M.E.) held in Cetraro, Italy, June 23–28, 1997 325–432 (2006).

