# Supplementary material for: Hierarchical modeling of mechano-chemical dynamics of epithelial sheets across cells and tissue
Source: Sci Rep. 2021 Feb 18;11:4069. doi: 10.1038/s41598-021-83396-6 (PMC7892579; doi:10.1038/s41598-021-83396-6)
Supplement: Supplementary file 1 — Supplementary Information. [file 41598_2021_83396_MOESM1_ESM.pdf]

Supplementary Information

Hierarchical modeling of mechano-chemical dynamics of epithelial sheets across cells and tissue

Yoshifumi Asakura[1], Yohei Kondo[2, 3, 4], Kazuhiro Aoki[2, 3, 4] and Honda Naoki[1, 5, 6*]

[1]Laboratory of Theoretical Biology, Graduate School of Biostudies, Kyoto University, Sakyo, Kyoto, Kyoto 606-8315, Japan
[2]Quantitative Biology Research Group, Exploratory Research Center on Life and Living Systems (ExCELLS), National Institutes of Natural Sciences, 5-1 Higashiyama, Myodaiji-cho, Okazaki, Aichi 444-8787, Japan.
[3]Division of Quantitative Biology, National Institute for Basic Biology, National Institutes of Natural Sciences, 5-1 Higashiyama, Myodaiji-cho, Okazaki, Aichi 444-8787, Japan.
[4]Department of Basic Biology, School of Life Science, SOKENDAI (The Graduate University for Advanced Studies), 5-1 Higashiyama, Myodaiji-cho, Okazaki, Aichi 444-8787, Japan.
[5]Research Center for Dynamic Living Systems, Kyoto University, Sakyo, Kyoto, Kyoto 606-8315, Japan
[6]Theoretical Biology Research Group, Exploratory Research Center on Life and Living Systems (ExCELLS), National Institutes of Natural Sciences, Okazaki, Aichi 444-8787, Japan.

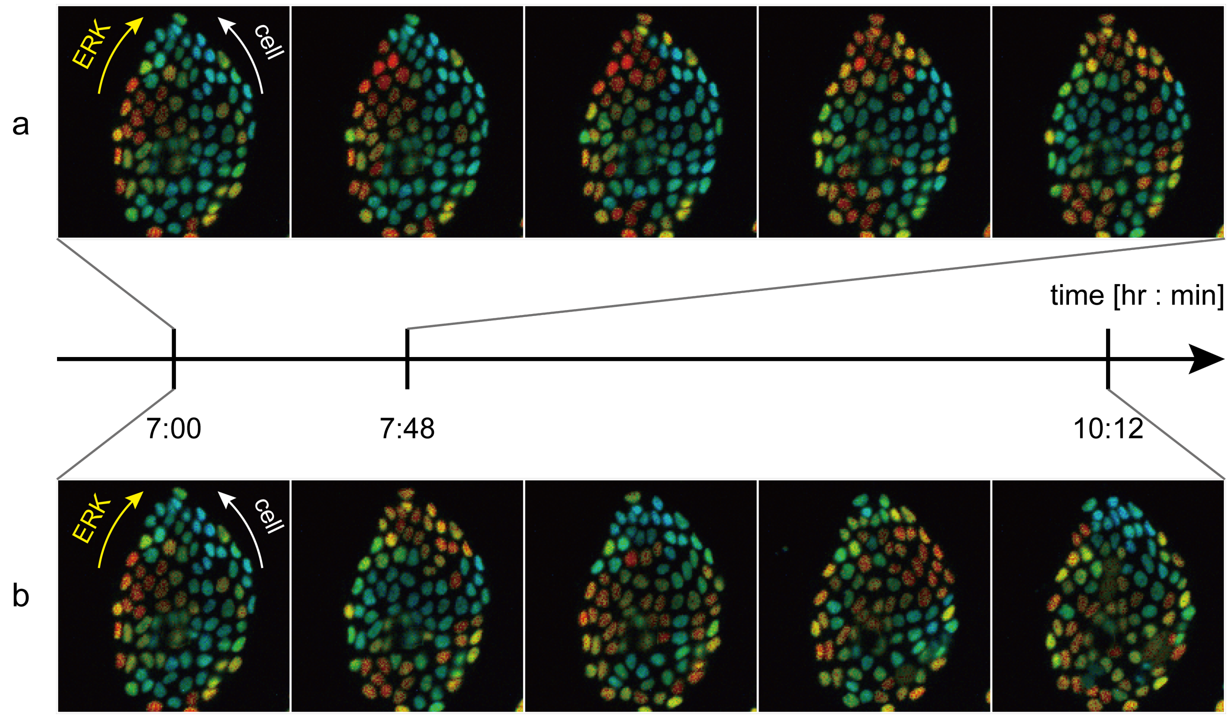

Supplemental figure 1: Clockwise rotation of ERK activation on a small cell cluster

a. Clockwise rotation of ERK activation waves in a shorter time range. Snapshots at every 12 minutes were shown here. Time coordinate corresponds to that in supplemental movie 1.

b. Counterclockwise rotation of cell migration in a longer time range. Snapshots in every 48 minutes were shown here for a clear visualization of slower cell migration than ERK wave propagation.

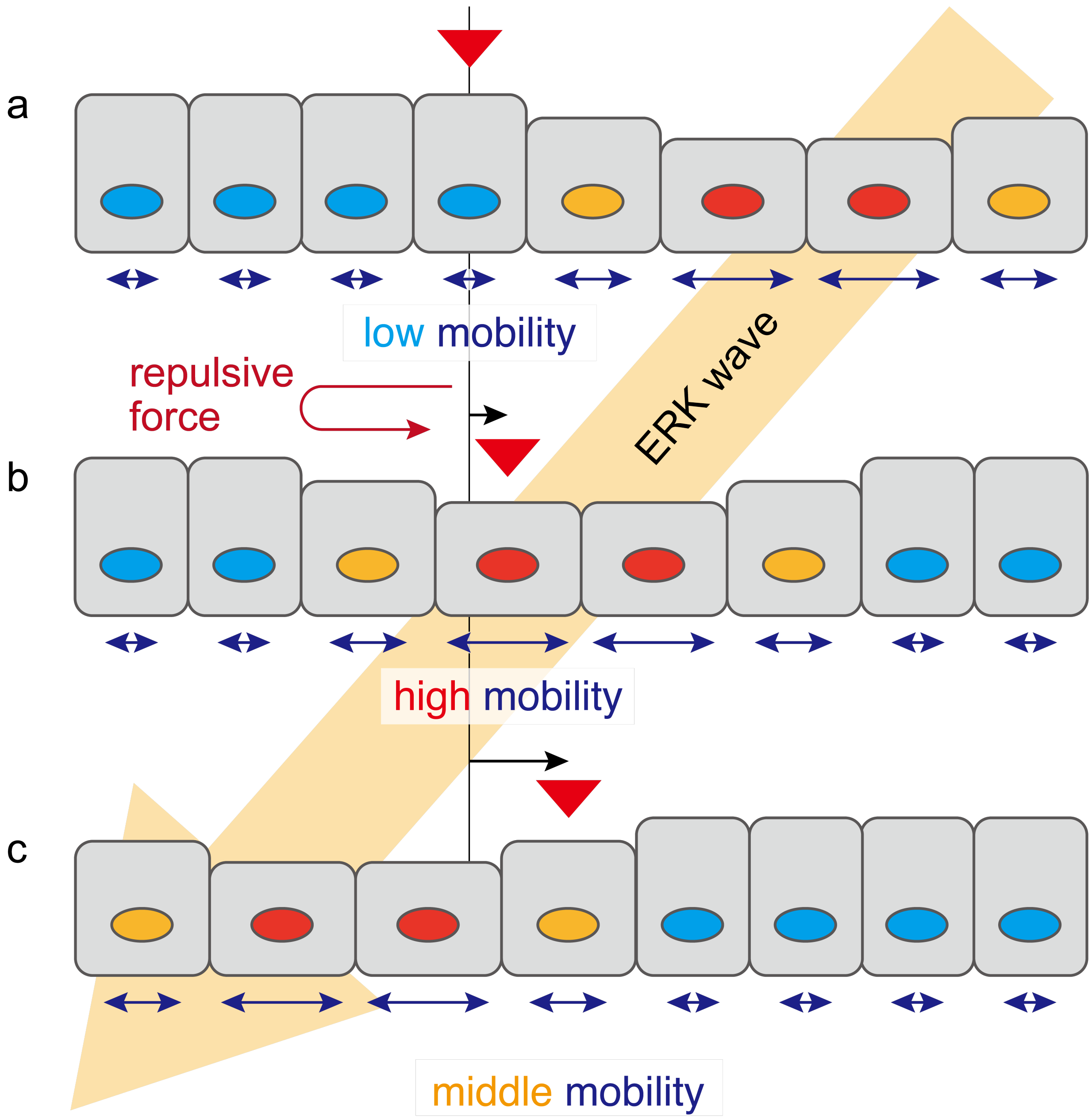

Supplemental figure 2: Schematic understanding of ERK-wave induced cell migration in the particle-based model

a. When the ERK wave is approaching, a cell indicated by a red triangle shows a low mobility because of low ERK activity but slightly moves left because right side cells with high ERK activity expand their volume and generate the pushing force.

b. When the ERK wave reached, the cell indicated by a red triangle shows a high mobility because of high ERK activity. ERK activity also induces the volume expansion and generates a pushing force to neighboring cells. The left side cells with low ERK activity resist the pushing force because of their low mobility and generate a repulsive force.

c. After a passage of ERK wave, left side cells cause a pushing force in the same manner as the cell indicated by a red triangle in (b), and the cell indicated by a red triangle is moved right.

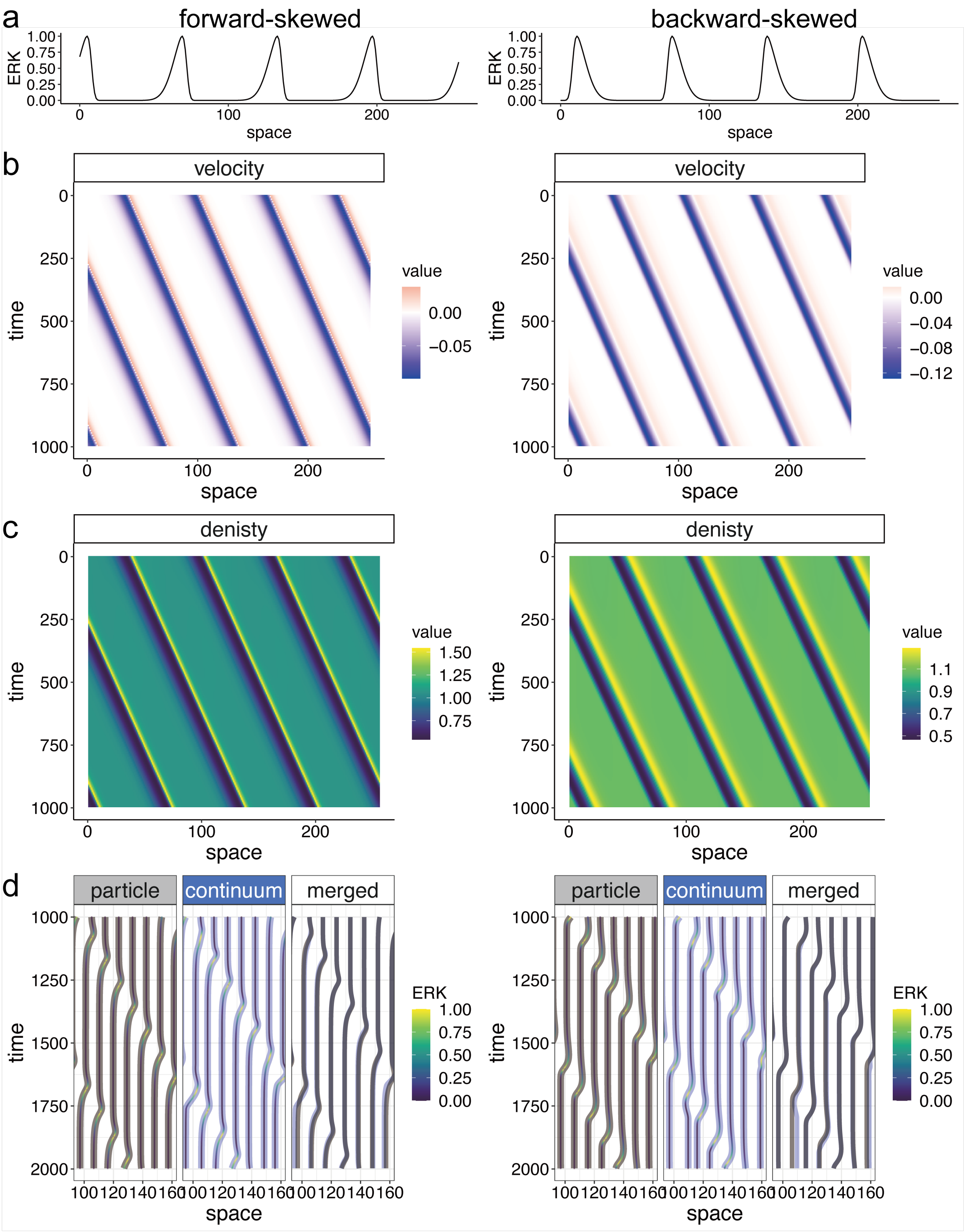

Supplemental figure 3: Simulations with asymmetric ERK waves

a. ERK waves in forward-skewed (left) and backward-skewed (right) shapes.

b - d. Simulated results with asymmetric ERK waves for velocity (b), density (c), and trajectories in the particle and continuum models (d).

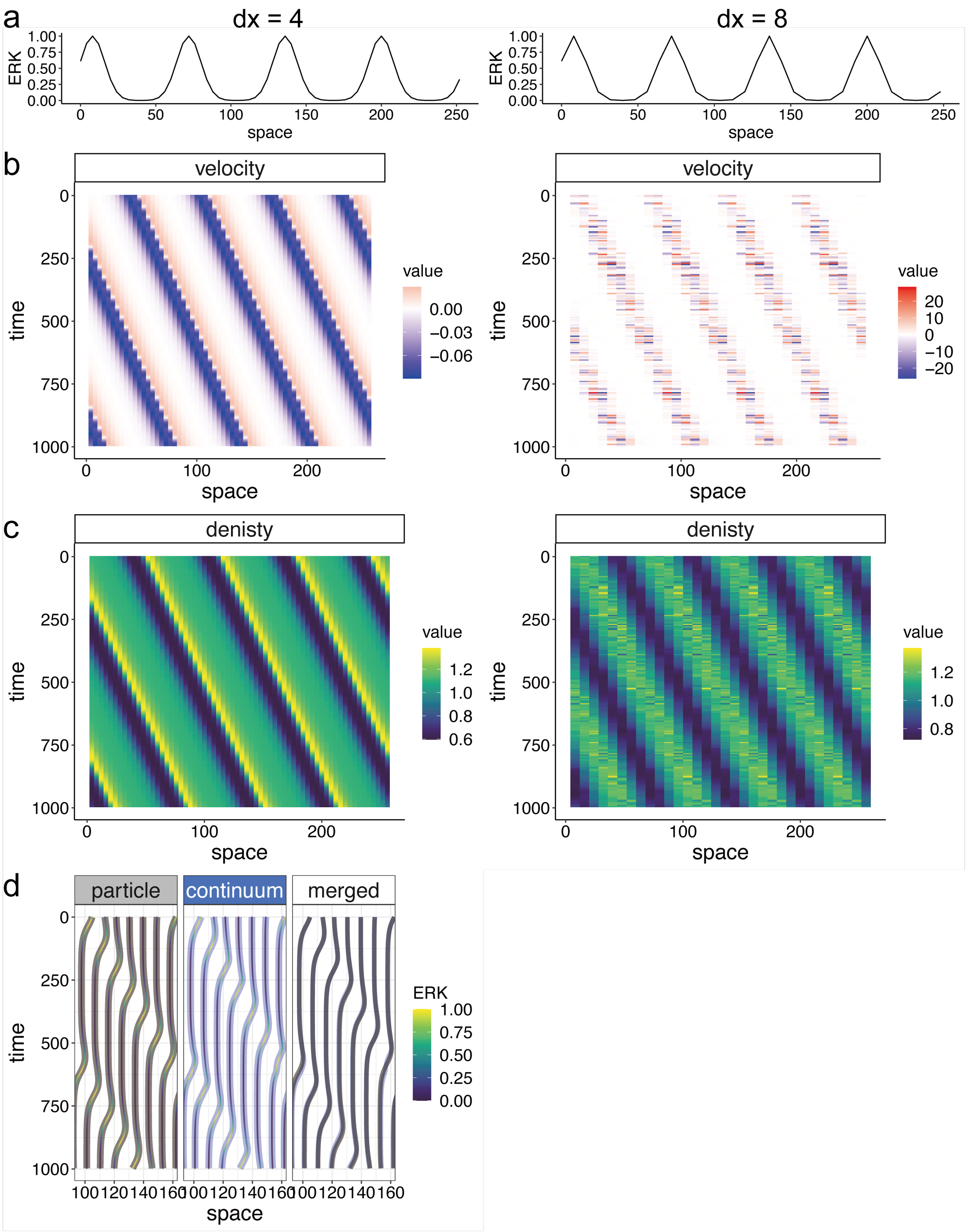

Supplemental figure 4: Simulations with larger gird sizes

a. ERK waves on different grid sizes. 4 (left) and 8 (right).

b - d. Simulated results for velocity (b), density (c), and trajectories in particle and continuum models (d). In (d), result on the right was omitted because of large numerical error.

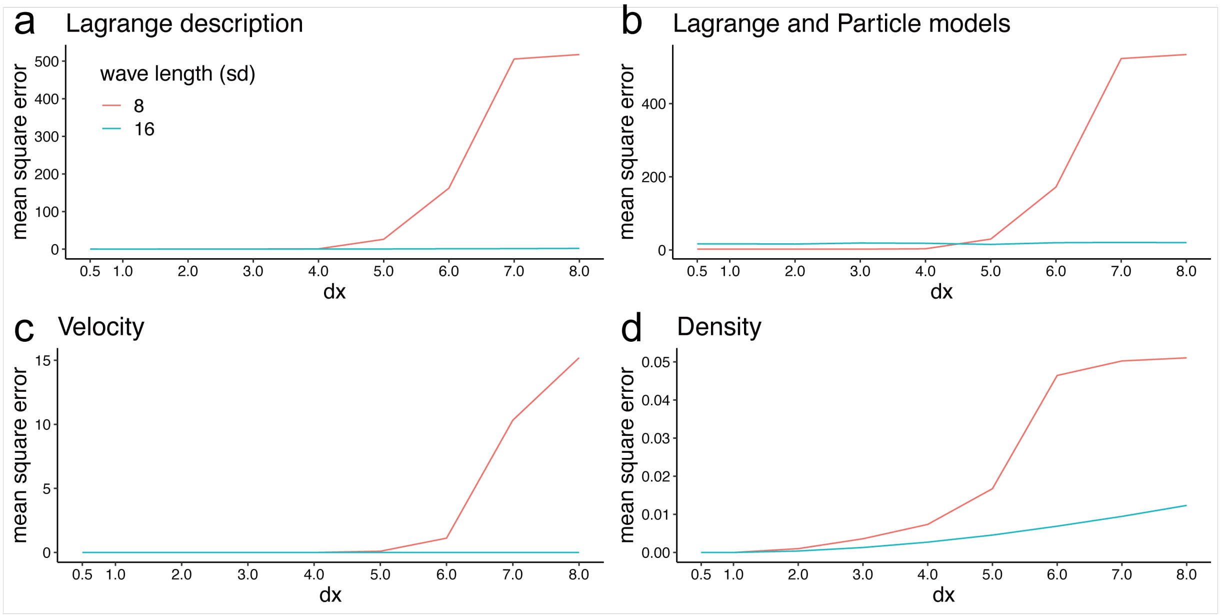

Supplemental figure 5: Effects of grid size on simulation

a. Mean square errors between simulated trajectories with various grid sizes and those in the case of unit grid size in the continuum model. Line colors indicate different width of ERK wave, i.e., a standard deviation (sd) of ERK Gaussian function.

b. Mean square errors between simulated trajectories with various grid sizes the continuum model and those in the particle-based model.

c. Mean square errors between simulated velocity values in Euler description with various grid sizes and those in the case of unit grid size in the continuum model.

d. Mean square errors between simulated density values in Euler description with various grid sizes and those in the case of unit grid size in the continuum model.

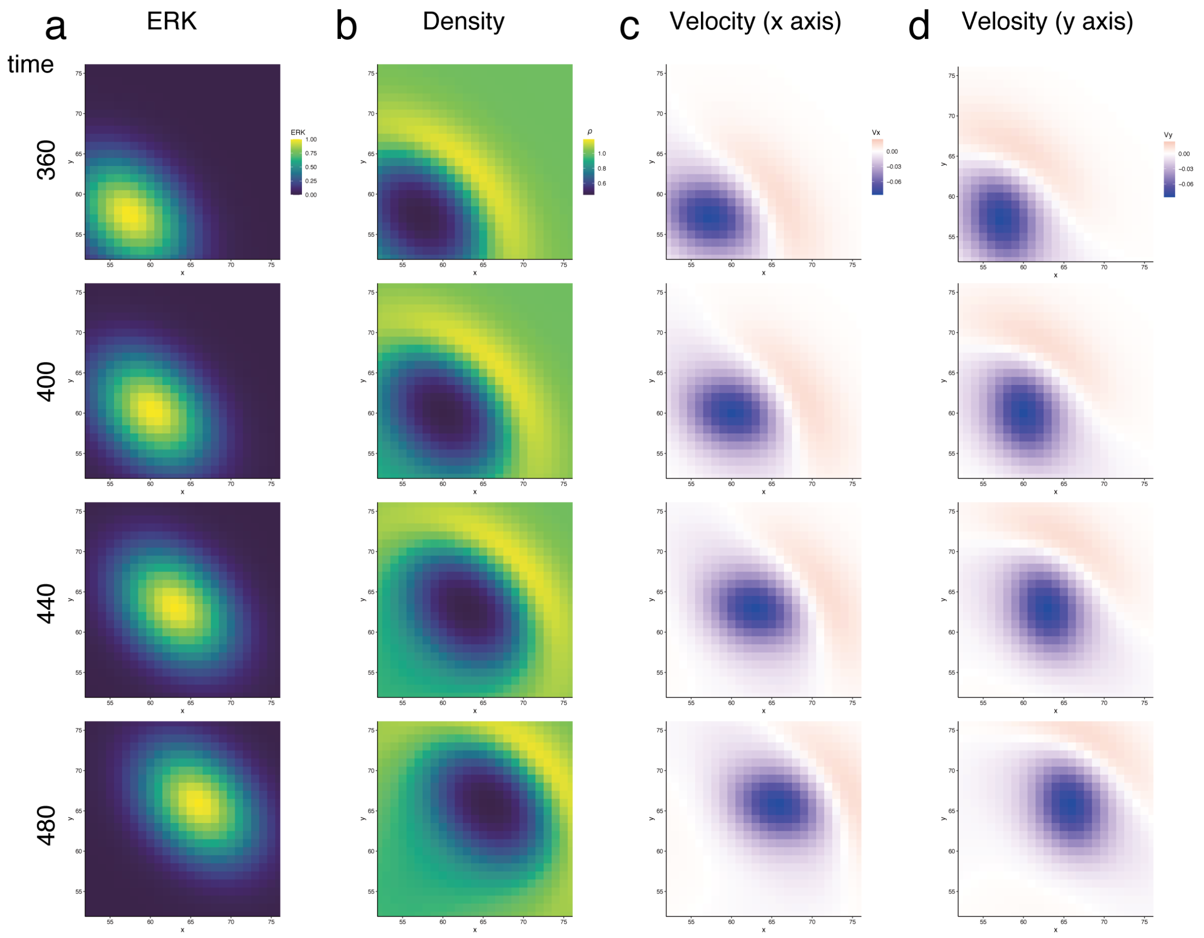

Supplemental figure 6: Two-dimensional simulation in the continuum model

a-d. Simulated results in Euler description. ERK wave (a), Cell density (b), Velocities on x-direction (c) and y-direction (d). See a supplemental movie for the simulation.

Supplementary movie legends

Supplemental movie 1. Live imaging of rotating dynamics of ERK waves and cell migration. Clockwise rotating ERK waves were associated with counterclockwise rotating cell migration, showing that cells migrate towards an opposite direction of ERK waves.

Supplemental movie 2. Simulated results in particle and continuum models. ERK wave (upper left), cellular migration in particle model (upper middle) and continuum model (upper right), cellular density (bottom left), velocity in x axis (bottom middle), and in y axis (bottom right).
